# Altered Fecal Microbiome and Metabolome in a Mouse Model of Choroidal Neovascularization

**DOI:** 10.3389/fmicb.2021.738796

**Published:** 2021-08-26

**Authors:** Yun Li, Yuting Cai, Qian Huang, Wei Tan, Bingyan Li, Haixiang Zhou, Zicong Wang, Jingling Zou, Chun Ding, Bing Jiang, Shigeo Yoshida, Yedi Zhou

**Affiliations:** ^1^Department of Ophthalmology, The Second Xiangya Hospital, Central South University, Changsha, China; ^2^Hunan Clinical Research Center of Ophthalmic Disease, Changsha, China; ^3^Department of Ophthalmology, Kurume University School of Medicine, Kurume, Japan

**Keywords:** choroidal neovascularization, age-related macular degeneration, gut microbiome, metabolomics, mouse model

## Abstract

**Purpose:**

Choroidal neovascularization (CNV) is the defining feature of neovascular age-related macular degeneration (nAMD). Gut microbiota might be deeply involved in the pathogenesis of nAMD. This study aimed to reveal the roles of the gut microbiome and fecal metabolome in a mouse model of laser-induced CNV.

**Methods:**

The feces of C57BL/6J mice with or without laser-induced CNV were collected. Multi-omics analyses, including 16S rRNA gene sequencing and untargeted metabolomics, were conducted to analyze the changes in the gut microbial composition and the fecal metabolomic profiles in CNV mice.

**Results:**

The gut microbiota was significantly altered in CNV mice. The abundance of *Candidatus_Saccharimonas* was significantly upregulated in the feces of CNV mice, while 16 genera, including *Prevotellaceae_NK3B31_group*, *Candidatus_Soleaferrea*, and *Truepera*, were significantly more abundant in the controls than in the CNV group. Fecal metabolomics identified 73 altered metabolites (including 52 strongly significantly altered metabolites) in CNV mice compared to control mice. Correlation analysis indicated significant correlations between the altered fecal metabolites and gut microbiota genera, such as *Lachnospiraceae_UCG-001* and *Candidatus_Saccharimonas*. Moreover, KEGG analysis revealed six pathways associated with these altered metabolites, such as the ABC transporter, primary bile acid biosynthesis and steroid hormone biosynthesis pathways.

**Conclusion:**

The study identified an altered fecal microbiome and metabolome in a CNV mouse model. The altered microbes, metabolites and the involved pathways might be associated with the pathogenesis of nAMD.

## Introduction

Age-related macular degeneration (AMD) is one of the main causes of vision loss and blindness worldwide, and its incidence has dramatically increased in the population worldwide (Mitchell et al., 2018). The presence of choroidal neovascularization (CNV) is the defining feature of wet or neovascular AMD (nAMD), which is one of the two advanced forms of AMD (Patel and Sheth, 2021). As a first-line therapy, intravitreal injection of anti-vascular endothelial growth factor (VEGF) agents is effective in patients with nAMD because it targets pathological CNV (Kovach et al., 2012; Ferrara and Adamis, 2016). However, the limitations of anti-VEGF therapy should not be ignored, such as the side effects of the injection (Xi, 2020) and the unsatisfactory duration of the therapeutic effect (Ehlken et al., 2019). In addition, long-term use of anti-VEGF therapy may lead to serious economic burdens, especially in developing countries and regions (Ruiz-Moreno et al., 2021). Therefore, thorough investigation of the mechanisms of nAMD pathogenesis beyond VEGF is urgently needed.

As an *in vivo* model, laser-induced CNV in mice is widely used to investigate the mechanisms of nAMD (Lambert et al., 2013). We have previously reported the expression profiles of mRNA and various types of non-coding RNAs in a CNV mouse model (Zhang et al., 2019, 2020; Liu et al., 2020) and indicated the importance of inflammatory cytokines and immune cells in AMD pathogenesis (Zhou Y. et al., 2017; Zhou Y. D. et al., 2017; Li and Zhou, 2019; Tan et al., 2020).

Changes in the intestinal microbiota significantly affect barrier function and metabolic pathways and gradually regulate the host immune system (Cerf-Bensussan and Gaboriau-Routhiau, 2010), and loss of gut microbiota diversity affects age-related changes (O’Toole and Jeffery, 2015). Recently, numerous studies have indicated that the gut microbiome is involved in ophthalmic diseases, such as diabetic retinopathy (DR) (Huang et al., 2021), Vogt-Koyanagi-Harada disease (Ye et al., 2020), and glaucoma (Gong et al., 2020).

Zinkernagel et al. (2017) revealed enrichment of the genera *Anaerotruncus* and *Oscillibacter* as well as *Ruminococcus torques* and *Eubacterium ventriosum* in nAMD patients; on the other hand, *Bacteroides eggerthii* was enriched in controls compared to patients. However, an intestinal metagenomic study with a larger number of included cases demonstrated elevated abundance of the class *Negativicutes* in patients with nAMD, while the genus *Oscillibacter* and *Bacteroides* species were more abundant in healthy controls without AMD (Zysset-Burri et al., 2020). Therefore, further explorations and verifications in more research centers are necessary to identify changes in the gut microbiota in AMD patients.

Overweight and obesity are essential risk factors for AMD (Zhang et al., 2016). High-fat diets (HFD) enhance pathology by inducing gut microbiota alteration, and the heightened intestinal permeability and chronic low-grade inflammation induced by gut dysbiosis have been found to upregulate the production of proinflammatory cytokines and VEGF-A and enhance CNV in a laser-induced mouse model (Andriessen et al., 2016). Therefore, alteration of the gut microbiome might be a potential therapeutic target in patients with AMD. Further investigation of the intestinal microbiome might reveal the mechanisms and metabolic pathways of AMD pathogenesis, which might also generate novel therapeutic strategies for AMD.

To clarify the pathogenesis and consequences of nAMD, in this study, we characterized fecal microbiome and metabolomics profiles in a mouse model of laser-induced CNV via 16S rRNA gene sequencing and untargeted metabolomics analysis.

## Materials and Methods

### Animal Model

Seven-week-old male C57BL/6J mice were obtained from Hunan SJA Laboratory Animal Co., Ltd. (Changsha, China). A model of CNV in the mice was induced by laser photocoagulation as described previously (Zhang et al., 2019). Laser photocoagulation was conducted with a 532-nm diode laser (100 mW, 0.1 s duration, 50 μm), with 25 spots burned on each eye.

Fecal samples were collected 7 days after laser photocoagulation. For control group, we used age-matched mice without laser treatment. Samples were collected from 16 mice with laser-induced CNV and 15 controls. The fecal pellets of each mouse were deposited into a sterile conical tube and stored at −80°C.

The animal experiments were performed according to the ARVO Statement for the Use of Animals in Ophthalmic and Vision Research, and the Institutional Animal Care and Use Committee of The Second Xiangya Hospital of Central South University approved all procedures of the experiments (Approval No. 2021533).

### DNA and Metabolite Extraction

DNA was isolated from fecal samples by using an E.Z.N.A.^®^ Soil DNA Kit (Omega Bio-Tek, Inc., Norcross, GA, United States). Assessment of the DNA extract was performed on an agarose gel (1%), and the concentration and purity of the DNA were determined by using a NanoDrop 2000 UV-Vis spectrophotometer (Thermo Fisher Scientific, Wilmington, DE, United States).

A 400 μL methanol:water (4:1, v/v) solution was used for the extraction of the fecal metabolites. The mixture was allowed to settle at −20°C. It was then treated with a Wonbio-96c high-throughput tissue crusher (Shanghai Wanbo Biotechnology Co., Ltd., Shanghai, China) at 50 Hz for 6 min, vortexed for 30 s and ultrasonicated at 40 kHz for 30 min. To precipitate proteins, the samples were placed at −20°C for 30 min. After centrifugation (13000 × *g*, 4°C, 15 min), the supernatant was collected for LC-MS/MS analysis.

### 16S rRNA Gene Sequencing Analysis

As previously described (Peng et al., 2018, 2019), the V3–V4 region of the bacterial 16S rRNA gene was amplified with the primers 338F (5′-ACTCCTACGGGAGGCAGCAG-3′) and 806R (5′-GGACTACHVGGGTWTCTAAT-3′) in an ABI GeneAmp^®^ 9700 PCR thermocycler (ABI, CA, United States). The purified amplicons were pooled in equimolar amounts and subjected to paired-end sequencing on an Illumina MiSeq PE300 platform/NovaSeq PE250 platform (Illumina, San Diego, CA, United States). To minimize the effects of sequencing depth on diversity measures, the number of reads from each sample was rarefied to 5567 (the minimum number of sample sequences). The Wilcoxon rank-sum test was used for statistical analysis of 16S rRNA gene sequencing analysis. The different enrichment of specific bacterial taxa was determined by the linear discriminant analysis (LDA) effect size (LEfSe) algorithm with an LDA score threshold of 2.0.

### Fecal Metabolomics Analysis

A Thermo UHPLC system equipped with an ACQUITY UPLC HSS T3 (100 mm × 2.1 mm i.d., 1.8 μm; Waters Corporation, Milford, MA, United States) was used for chromatographic separation of the metabolites. A Thermo UHPLC-Q Exactive Mass Spectrometer equipped with an electrospray ionization (ESI) source operating in either positive or negative ion mode was used to collect the mass spectrometric data. Data-dependent acquisition (DDA) mode was used for the data acquisition. Detection was conducted over the mass range of 70–1050 m/z.

After UPLC-MS analyses, the raw data were imported into Progenesis QI 2.3 (Non-linear Dynamics, Waters Corporation, United States) for peak detection and alignment. The mass spectra of these metabolic features were identified by using the accurate masses, MS/MS fragment spectra and isotope ratio differences with searching in the following biochemical databases: the Human Metabolome Database (HMDB)^1^ and the METLIN database^2^.

### Multivariate Statistical Analysis

Variables of all metabolites were scaled to unit variances and then subjected to principal component analysis (PCA) to obtain a visualized overview of the metabolic data, general clustering, trends, and outliers. Orthogonal partial least squares discriminant analysis (OPLS-DA) was used to determine the global alterations of metabolites between the CNV group and the control group. Prior to OPLS-DA, all of the metabolite variables were Pareto-scaled. Variable importance in the projection (VIP) was calculated from the OPLS-DA model. Paired Student’s *t*-test was used in calculating *P*-values. Statistically significant differences between CNV group and control group were determined according to *p* < 0.05 and VIP > 1.0.

### Bioinformatics Analyses

The Majorbio I-Sanger Cloud Platform^3^ was used for the data analyses and bioinformatics analyses. The pathways associated with the altered metabolites were analyzed through metabolic enrichment and pathway analyses according to a database search (KEGG)^4^. Spearman’s correlation analysis was conducted to assess the significance of microbiota-metabolite correlations with the threshold values of | *r*| ≥ 0.50 and *p* < 0.01.

## Results

### Diversity of the Gut Microbiota Between CNV Mice and Controls

**FIGURE 1:**
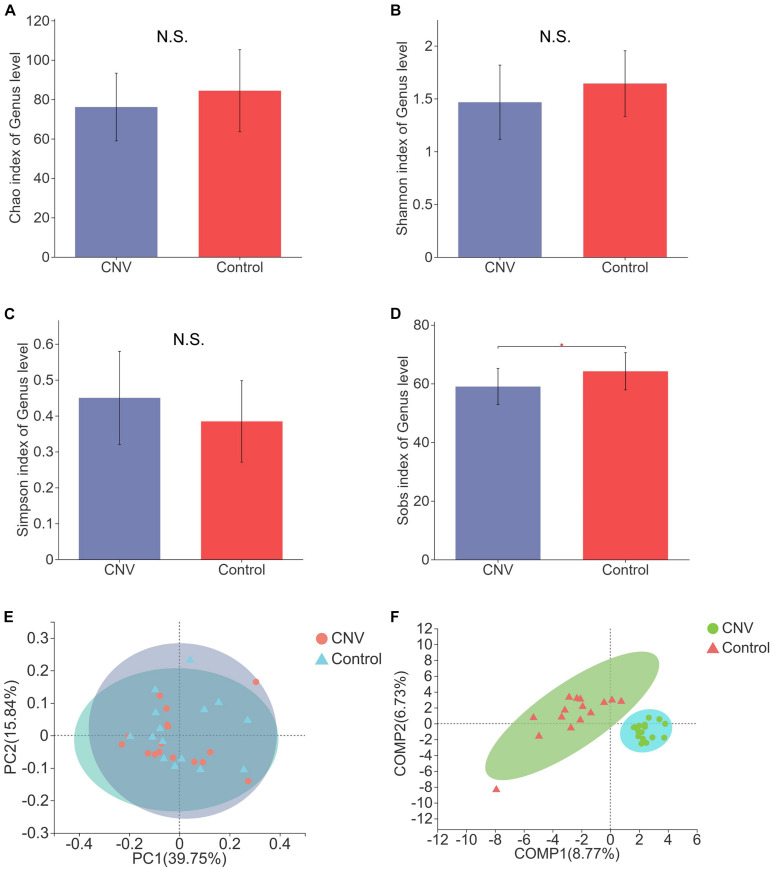
Gut microbial diversity in CNV and control mice. Gut microbial diversity in CNV and control mice. The α-diversity was assessed by the Chao index **(A)**, Shannon index **(B)**, Simpson index **(C)**, and Sobs index **(D)**. ^∗^*P* < 0.05. PCoA of the β-diversity **(E)**. PLS-DA of the microbiome at the genus level **(F)**.

To reveal the differences in structural diversity of the gut microbiota between CNV mice and controls, microbial α-diversity was assessed using the Chao, Shannon, Simpson, and Sobs indices. Although no significant difference in α-diversity was observed by measurement of the Chao, Shannon, and Simpson indices (Figures 1A–C, *p* > 0.05), significantly lower diversity was found in the CNV group than in the control group, as measured by the Sobs index (Figure 1D, *p* = 0.03079). For the β-diversity analysis, principal coordinate analysis (PCoA) was used after a genus selection-based bacterial taxonomy analysis was performed, and significant differences were not observed when the CNV group was compared with the control group (Figure 1E). However, PLS-DA indicated that the samples derived from the CNV group significantly differed from those collected from the control group (Figure 1F), which demonstrated the different compositions of the gut microbiota between these two groups.

### Change in the Gut Microbiota Composition in CNV Mice

**FIGURE 2:**
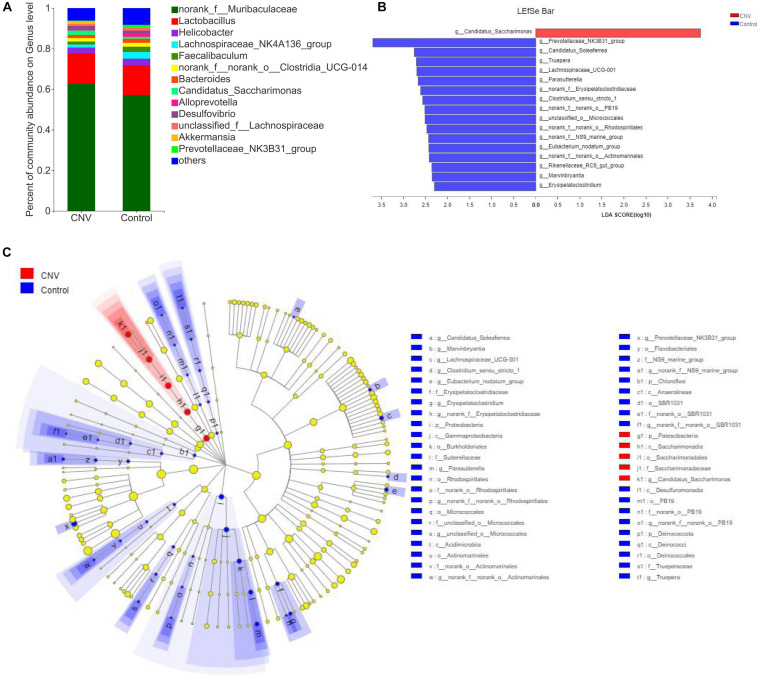
Gut microbiota composition profiles in CNV and control mice. **(A)** Taxonomic distributions of bacteria at the genus level in CNV mice and controls. **(B)** Bar graph of LDA scores to screen altered bacterial genera at the genus level (LDA score ≥2.0). **(C)** Cladogram of the LEfSe analysis from the phylum level to the genus level of the microbiota of CNV mice and controls.

Taxonomic analysis revealed the differences in relative abundance at the genus level between CNV and control mice (Figure 2A). Among the genera, *norank_f_Muribaculaceae* was the predominant genus in both the CNV group (62.7%) and the control group (57.2%). By the LEfSe algorithm, we identified 17 genera as key discriminants (Figures 2B,C). *Candidatus_Saccharimonas* was significantly overrepresented in the feces of CNV mice, while 16 genera, including *Prevotellaceae_NK3B31_group*, *Candidatus_Soleaferrea*, and *Truepera*, were significantly more abundant in the control group than in the CNV group. These results demonstrate the different fecal microbiota compositions between these two groups.

### Altered Fecal Metabolomic Profiles of CNV Mice

**FIGURE 3:**
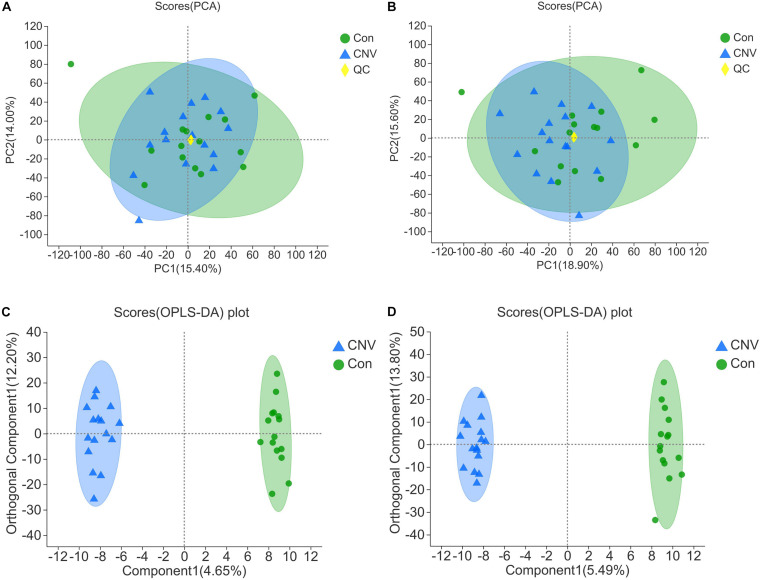
Qualification of the results of fecal metabolomic analysis. PCA of all the samples under the positive **(A)** and negative ion mode **(B)**. OPLS-DA score plots of CNV mice and controls under the positive **(C)** and negative ion mode **(D)**.

The fecal samples above were also used for identification of metabolites that are altered in CNV by metabolomics. The QC samples clustered closely in both positive and negative ion modes in the PCA (Figures 3A,B). OPLS-DA score plots revealed remarkable separation of these two groups under both modes (Figures 3C,D).

**FIGURE 4:**
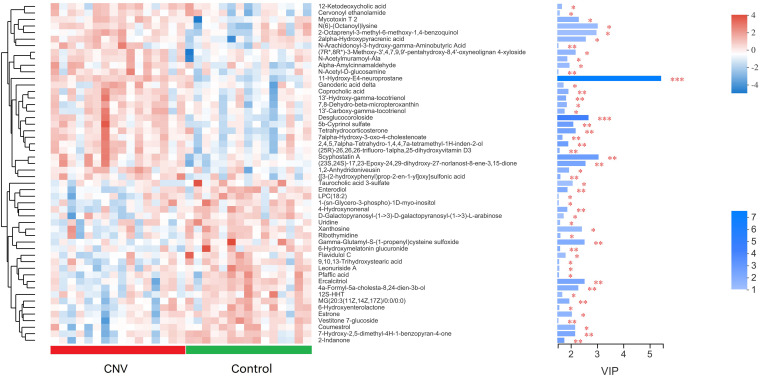
Metabolomic profiles of fecal samples from CNV mice and controls. The hierarchical cluster and heat map show the 52 strongly significantly altered metabolites in each sample. Red and blue colors represent high and low levels of metabolites, respectively. The bar graph shows the VIP scores of each metabolite. ^∗^*p* < 0.05, ^∗∗^*p* < 0.01, ^∗∗∗^*p* < 0.001.

Metabolites with *p* < 0.05 and VIP > 1 were considered to be significantly altered (Supplementary Table 1), and those with *p* < 0.05 and VIP > 1.5 were considered to be strongly significantly altered (Supplementary Table 2). In total, 73 significantly altered metabolites (24 in positive ion mode and 49 in negative ion mode) and 52 strongly significantly altered metabolites (21 in positive ion mode and 31 in negative ion mode) were identified between the CNV and control groups. To visualize these 52 strongly significantly altered metabolites, we constructed a heat map (Figure 4). Overall, 27 metabolites were significantly increased in CNV mice, while 25 metabolites were significantly decreased in CNV mice. Among them, 25 metabolites belonged to the superclass of lipids and lipid-like molecules, which accounted for the largest proportion of the strongly significantly altered metabolites.

### Correlations of the Fecal Metabolome and Gut Microbiota

**FIGURE 5:**
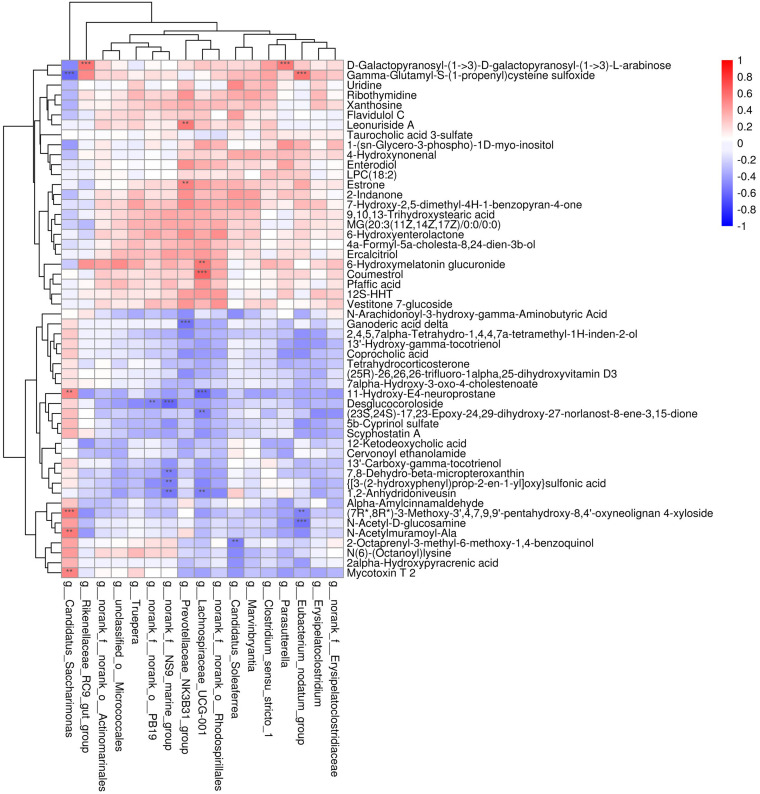
Correlation analysis between the discriminated gut microbiota and the strongly altered fecal metabolites. Red and blue colors represent positive and negative correlations, respectively. Significant correlations were determined according to the threshold values of | *r*| > 0.5 and *P* < 0.01. **p < 0.01, ***p < 0.001.

To explore the functional correlations between the alterations of the gut microbiome and the fecal metabolome, Spearman’s correlation coefficient analysis was conducted between the 17 discriminatory genera and 52 strongly significantly altered metabolites (*p* < 0.05 and VIP > 1.5). A total of 24 significant correlations were recognized (Figure 5). In particular, both *Lachnospiraceae_UCG-001* and *Candidatus_Saccharimonas* were significantly associated with five fecal metabolites. Moreover, *norank_f__NS9_marine_group*, *Prevotellaceae_NK3B31_group*, and *Eubacterium_nodatum_group* were significantly associated with 4, 3, and 3 metabolites, respectively. The correlations indicated that CNV mice demonstrated significant alterations in their gut microbiomes that may have led to significant changes in their metabolomic profiles.

### Pathways Associated With the Altered Fecal Metabolites According to KEGG Analysis

**FIGURE 6:**
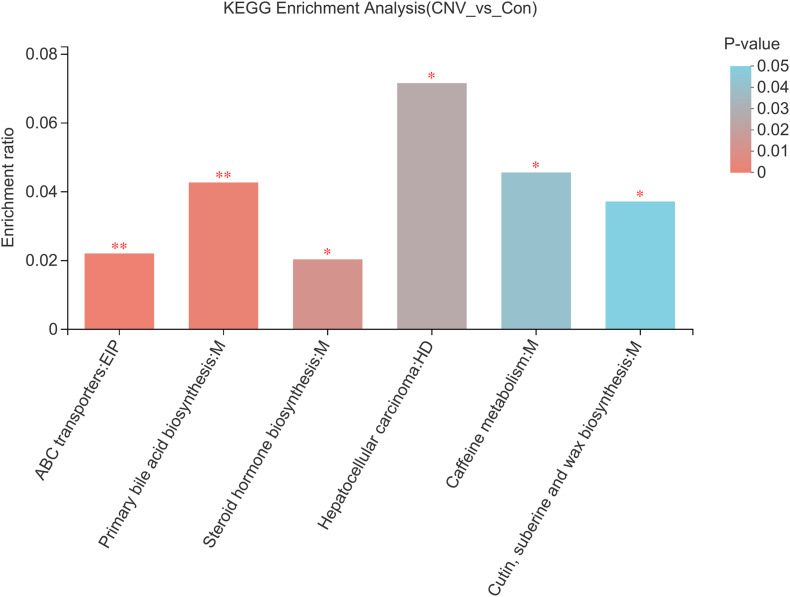
KEGG pathway analysis reveals the pathways associated with the altered fecal metabolites. Colors represent the sizes of the *p*-values. ^∗^*p* < 0.05, ^∗∗^*p* < 0.01.

To identify the pathways associated with these metabolites, KEGG pathway enrichment analyses were performed for the 73 significantly altered metabolites. Several essential pathways were detected (*p* < 0.05), as follows: the ATP-binding cassette (ABC) transporter pathway; primary bile acid biosynthesis; steroid hormone biosynthesis; hepatocellular carcinoma; caffeine metabolism; and cutin, suberine, and wax biosynthesis (Figure 6).

## Discussion

In this study, we characterized the gut microbiome and fecal metabolome in mice with laser-induced CNV, a widely used model of nAMD. The results indicated that the composition of the gut microbiota and the levels of fecal metabolites were significantly altered in CNV mice compared to the age-matched controls.

Linear discriminant analysis effect size revealed *Candidatus_Saccharimonas* as the only dominant genus in the CNV group, while we found 16 genera that were more abundant in the control group than in the CNV group (Figure 2). The bacterial genus *Candidatus_Saccharimonas*, which belongs to the phylum *Patescibacteria* (Lemos et al., 2019), was upregulated in CNV mice compared with control mice. Chen et al. (2021) revealed that the probiotic LPPS23 enriches *Candidatus_Saccharimonas* in aged mice. Green tea leaf powder improves lipid metabolism in HFD-fed mice, and gut microbiota reprogramming might be involved in the mechanism. Green tea leaf powder reduces systemic inflammation and the abundance of *Candidatus_Saccharimonas* in HFD-fed mice (Wang et al., 2020). Another study has demonstrated that egg white peptides significantly increase the relative abundance of *Candidatus_Saccharimonas* and inhibit the production of proinflammatory cytokines (Ge et al., 2021). Sang et al. (2020) reported that the mushroom *Bulgaria inquinans* reduces the diversity of the gut microbiota and downregulates the abundance of *Candidatus_Saccharimonas* and that *Candidatus_Saccharimonas* is positively correlated with several cytokines (IL-2, IL-4, IL-10, and IFN-γ). Therefore, despite some unclear mechanisms, *Candidatus_Saccharimonas* might be associated with inflammation and the host immunological response. As previously described, inflammation plays an essential role in the pathogenesis of AMD (Tan et al., 2020), and it is worth further clarifying the roles and mechanisms of *Candidatus_Saccharimonas* in inflammation associated with AMD pathogenesis in future studies.

In addition, we identified 73 metabolites that were altered in CNV mice compared to controls and found that 52 of them had strong significant alteration. KEGG analysis revealed six pathways associated with these altered metabolites, such as the ABC transporter pathway. ABC transporter A1 (ABCA1), a gene involved in high-density lipoprotein (HDL) metabolism, mediates the lipid efflux pathway and has functional effects in RPE cells, and it might also contribute to the development and progression of AMD (Storti et al., 2017). Storti and Grimm (2019) reported the essential role of the ABCA1/G1 pathway and the mechanism of active cholesterol efflux in the RPE, rods, and retinal inflammatory cells. Interestingly, in a study we have previously reported, the pathway of ABC transporters was also found to be associated with plasma metabolites that are altered in retinopathy of prematurity, which is another kind of ocular neovascular disease that occurs in premature infants (Zhou et al., 2020). Therefore, the association of mechanisms of ABC transporters with the gut microbiota in AMD needs to be further studied.

Significant correlations were observed between the altered fecal metabolites and gut microbiota genera such as *Lachnospiraceae_UCG-001* and *Candidatus_Saccharimonas*. *Lachnospiraceae UCG-001* produces short-chain fatty acids, and compositional alterations of gut microbiotas including this genera have been found to be associated with inhibition of colon inflammation and tumorigenesis (Guo and Li, 2019). The abundance of *Lachnospiraceae_UCG_001* is lower in rats with ischemic stroke than in sham rats (Wu et al., 2021). Additionally, opposite to the alteration in the abundance of *Candidatus_Saccharimonas*, the abundance of *Lachnospiraceae_UCG-001* is suppressed by the probiotic LPPS23 in aged mice (Chen et al., 2021). These findings indicate that the genera *Lachnospiraceae_UCG-001* and *Candidatus_Saccharimonas* together with their associated altered fecal metabolites might be involved in the pathogenesis of nAMD, which is worth further exploration.

Recent studies demonstrated the alterations and possible application prospects of the gut microbiome in patients with other ocular neovascular diseases, such as DR (Das et al., 2021; Huang et al., 2021) and retinopathy of prematurity (Skondra et al., 2020). Das et al. (2021) recognized a reduction in anti-inflammatory, probiotic and other bacteria that could be pathogenic in the microbiomes of patients with both diabetes mellitus and DR, compared to the healthy controls, and the changes observed in DR patients were more pronounced. Huang et al. (2021) indicated the potential use of gut microbiota as a biomarker of DR, which could be helpful for diagnosis in clinical applications. Moreover, it has been suggested that the effect of antihyperglycemic drugs might be involved in the connection between the gut microbiota and DR, and targeting the gut microbiome could be novel therapeutic strategies in treating DR (Rowan and Taylor, 2018).

There were some limitations of our present study. First, the laser-induced CNV model in mice cannot completely recapitulate the characteristics of clinical samples of nAMD patients; thus, larger cohorts of patients should be investigated in future studies. Second, this study included only one time point (day 7 after laser photocoagulation), which is a representative time point for CNV. However, it is still necessary to assess alterations of the gut microbiota and metabolomics at multiple time points, especially during the period of subretinal fibrosis, which is 3–4 weeks after laser photocoagulation. Third, the roles and regulatory functions of the altered gut microbes and fecal metabolites remain to be further studied. Fecal microbiota transplantation is a novel therapy to restore the gut microbiota and cure diseases, and the investigation of this field is rapidly emerging in many diseases (Vindigni and Surawicz, 2017). Andriessen et al. (2016) confirmed that fecal microbiota transplantation regulates pathological angiogenesis in obesity-driven CNV *in vivo*. Therefore, this method could be used in future studies to investigate the functions and mechanisms of gut microbes and fecal metabolites in CNV and nAMD.

In conclusion, we demonstrated significant alterations of the gut microbiome and fecal metabolome in CNV mice. Some altered gut microbe genera, such as *Lachnospiraceae_UCG-001* and *Candidatus_Saccharimonas*, were strongly correlated with altered fecal metabolites. Our results demonstrated concurrent alterations of the gut microbiota and fecal metabolites during the pathological process of CNV. Further studies are needed to reveal whether these altered microbiota and metabolites as well as their associated pathways play modulatory roles in CNV and nAMD pathogenesis, which might be helpful in developing novel therapeutic strategies of nAMD.

## Data Availability Statement

The raw data of 16S rRNA sequencing was deposited in NCBI Sequence Read Archive (SRA) (accession No. PRJNA744326).

## Ethics Statement

The animal study was reviewed and approved by the Institutional Animal Care and Use Committee of The Second Xiangya Hospital of Central South University.

## Author Contributions

YZ conceived and designed the study. YZ, YL, and YC wrote the manuscript. YZ, YL, YC, QH, WT, and BL performed the experiments and collected the samples. YZ, HZ, ZW, CD, and JZ analyzed the data. BJ and SY reviewed the manuscript. All authors read and approved the final manuscript.

## Conflict of Interest

The authors declare that the research was conducted in the absence of any commercial or financial relationships that could be construed as a potential conflict of interest.

## Publisher’s Note

All claims expressed in this article are solely those of the authors and do not necessarily represent those of their affiliated organizations, or those of the publisher, the editors and the reviewers. Any product that may be evaluated in this article, or claim that may be made by its manufacturer, is not guaranteed or endorsed by the publisher.

## Footnotes

1
www.hmdb.ca

2
metlin.scripps.edu

3
www.i-sanger.com

4
www.genome.jp/kegg/

## Supplementary Material

Supplementary Table 1 The significantly altered fecal metabolites (Vip > 1 and *p* < 0.05) between Cnv group and control group.

Supplementary Table 2 The strongly significantly altered fecal metabolites (Vip > 1.5 and *p* < 0.05) between Cnv group and control group.
